# Robotic Vectorial Field Alignment for Spin‐Based Quantum Sensors

**DOI:** 10.1002/advs.202304449

**Published:** 2023-11-17

**Authors:** Joe A. Smith, Dandan Zhang, Krishna C. Balram

**Affiliations:** ^1^ Quantum Engineering Technology Labs and Department of Electrical and Electronic Engineering University of Bristol Bristol BS8 1FD UK; ^2^ Bristol Robotics Laboratory and Department of Engineering Mathematics University of Bristol Bristol BS8 1TW UK

**Keywords:** NV centers, quantum technology, robotics, spin‐based sensors, vectorial sensing

## Abstract

Developing practical quantum technologies will require the exquisite manipulation of fragile systems in a robust and repeatable way. As quantum technologies move toward real world applications, from biological sensing to communication in space, increasing experimental complexity introduces constraints that can be alleviated by the introduction of new technologies. Robotics has shown tremendous progress in realizing increasingly smart, autonomous, and highly dexterous machines. Here, a robotic arm equipped with a magnet is demonstrated to sensitize an NV center quantum magnetometer in challenging conditions unachievable with standard techniques. Vector magnetic fields are generated with 1° angular and 0.1 mT amplitude accuracy and determine the orientation of a single stochastically‐aligned spin‐based sensor in a constrained physical environment. This work opens up the prospect of integrating robotics across many quantum degrees of freedom in constrained settings, allowing for increased prototyping speed, control, and robustness in quantum technology applications.

## Graphical abstract

Robotic arms consist of sets of links that allow arbitrary positioning and orientation of an end tool. This study shows such a technique can be used to align vector spin‐based quantum sensors with high accuracy by positioning a permanent magnet. The flexibility demonstrated is important for emerging quantum sensing applications in physically constrained environments.

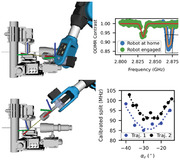

## 1 Introduction

Experiments designed to exploit quantum technologies for applications can be extremely challenging. Fragile quantum states must be delicately manipulated, whilst minimizing sources of decoherence, in order to preserve a quantum advantage. This often necessitates cutting‐edge experimental physics, including precise and complex optical assemblies,^[^ ^1^, ^2^ ^]^ strong vector magnetic fields,^[^ ^3^ ^]^ high‐speed microwave delivery,^[^ ^4^ ^]^ and compatibility with extremely low temperature environments.^[^ ^5^ ^]^ Emerging quantum technologies based on hybrid quantum systems^[^ ^6^ ^]^ combine research from two or more experimental settings: such as coupling spins in silicon to superconducting resonators and qubits,^[^ ^7^, ^8^ ^]^ interfacing remote NV centers in diamond with photonic qubits,^[^ ^9^ ^]^ and using nanomechanics to interface with spins^[^ ^10^ ^]^ or superconducting qubits.^[^ ^11^ ^]^

As these proof‐of‐principle devices become more sophisticated and start to scale in size and complexity, established lab infrastructure such as translation stages and solenoid coils will no longer provide the flexibility, speed, and precision to meet these constrained^[^ ^12^ ^]^ and sometimes competing experimental requirements. In contrast, the field of robotics has long adapted to operate robots in challenging conditions, such as at the microscale^[^ ^13^ ^]^ or in very low temperature environments.^[^ ^14^ ^]^ Robotics can provide more flexible and adaptable approaches than traditional methods, which would speed up the deployment of quantum technology across applications. With sophisticated software stacks and well‐developed open‐source hardware, the deployment of robotics in a diverse range of experimental settings in the chemical and biological sciences has become increasingly feasible.^[^ ^15^, ^16^ ^]^

**Figure 1:**
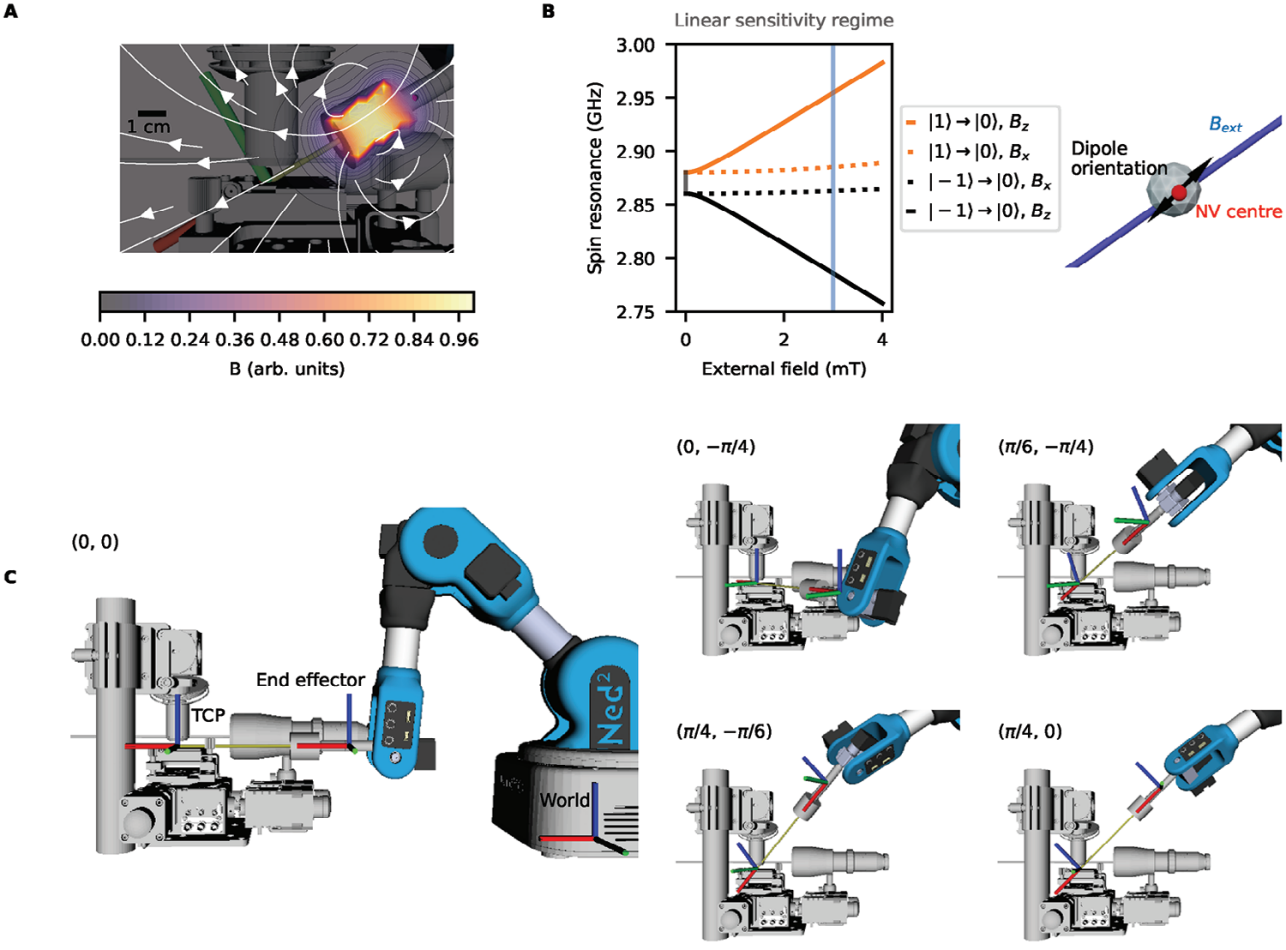
Experimental setup and working principles. A) Placing a permanent magnet near the NV center magnetometer produces a magnetic field of a known orientation, defined along its axis (field lines in white). B) One use case here is to change the spin resonance of the magnetometer to operate at its most sensitive regime (linear with respect to detected field) away from the zero‐field splitting (marked in gray). As observed, field along the NV center *B*_*z*_ affects this response, whereas transverse components only contribute unwanted performance degradation. The field *B*_ext_ should therefore be approximately aligned to the NV center magnetic dipole orientation *B*_*z*_. C) The 6 DoF robot is used to orient the magnet in complex surroundings. The robot base is located at the world origin (*x*‐axis indicated in red, *y*‐axis indicated in green, *z*‐axis in blue). The tool center point (TCP axis marked) is translated along the *x*‐axis of the end effector axis (marked) to set the required field strength. The TCP coordinates (*x*, *y*, *z*, α_*y*_, α_*z*_) are then set to the NV center position and rotated around the *y* and *z* axes, that is, varying α_*y*_ and α_*z*_ of the TCP, to form a defined vector from the end effector to the TCP (shown in yellow). The robot position at a range of different (α_*y*_, α_*z*_) is shown in the inset diagrams. Through this method, the highly‐dexterous robot can create fields with arbitrary field strengths and orientations and align the TCP axis with the NV axis to produce the desired *B*_*z*_.

Here, we introduce and validate the idea of robot‐assisted quantum technology. Specifically, we employ the use of a robotic arm to hold a strong permanent magnet to meet a requirement in spin‐based sensing: aligning an external magnetic field along the magnetic dipole axis of an arbitrarily oriented spin system (**Figure** **1**a). We demonstrate that this method has significant advantages where traditional techniques for generating vector fields, such as mounting the magnet on a fixed axis translation stage, or using a three‐axis Helmholtz coil, are infeasible owing to the tight physical constraints of the surrounding optomechanical apparatus. While this work focuses on a specific use case for robotics in quantum technology, the methods developed here can be easily adapted and extended to other experimental settings.

### 1.1 Problem Statement and Requirements

Spin‐based magnetometers operate by mapping local perturbations in their environment to shifts in the transition (magnetic resonance absorption) frequency of the spin system.^[^ ^17^ ^]^ The NV center in diamond, an atom‐like defect comprising a nitrogen atom and neighboring vacancy site,^[^ ^18^ ^]^ is the prototypical solid state quantum sensor on account of its optically accessible spin state, which allows optical manipulation and readout of its spin state at room temperature (optically detected magnetic resonance or ODMR). NV center magnetometers have rapidly advanced over the past decade and have reached maturity as a quantum magnetometer, with nanotesla (nT) sensitivities at nanoscale resolutions.^[^ ^19^, ^20^ ^]^ As magnetic dipole–dipole interactions are weak and confined to the near field, near‐surface NV centers are required to image fields from individual spins.^[^ ^21^ ^]^ Nanoscale inclusions of diamond, or nanodiamond, are used to host the spin probe in hot and wet biochemical surroundings in applications such as protein^[^ ^22^, ^23^ ^]^ or cell detection.^[^ ^24^, ^25^ ^]^ Nanodiamonds typically contribute an additional energy term Π to the NV center Hamiltonian *H*, from lattice strain and local charges^[^ ^26^, ^27^ ^]^

(1) $$\begin{array}{c} H=DS_{z}^{2}+\Pi \left(S_{x}^{2}-S_{y}^{2}\right)+\gamma B_{⊥}\cdot S_{⊥}+\gamma B_{z}S_{z} \end{array}$$

where **B**_⊥_ = (*B*_*x*_, *B*_*y*_) and **S**_⊥_ = (*S*_*x*_, *S*_*y*_) are the transverse magnetic field and Pauli spin terms, with *z* defined as the axis comprising the NV center along the diamond lattice. In Figure 1b, the energy term Π leads to a frequency splitting of size 2Π (shown in gray), making the NV center transition frequencies robust to magnetic field fluctuations to first order. A bias field *B*_*z*_ is thus required bring to the NV center into the regime (*B*_*z*_ ≫ Π/γ) where the transitions are linearly dependent on the magnetic field, which corresponds to the highest sensitivity. Given that nanodiamonds typically display Π ≈ 10 MHz,^[^ ^28^ ^]^ this requires a moderate *B*_*z*_ magnetic field of 5 mT aligned to the NV axis. A misaligned magnetic field (with a residual *B*_*x*_ or *B*_*y*_ component) would lead to a mixing of the energy eigenstates, which would result in a reduction of both the fluorescence and contrast (SNR) of the spin‐dependent optical readout.^[^ ^29^ ^]^ Magnetic fields of 5 mT significantly degrade the spin coherence time (*T*_2_) of the NV center when misaligned by 5° as they cause nearby nuclear spins to precess.^[^ ^30^, ^31^ ^]^

To date, the established method to align a static magnetic field to an arbitrarily oriented spin is using three perpendicular wire coils^[^ ^32^, ^33^, ^34^ ^]^ or sets of coils in the Helmholtz configuration.^[^ ^28^, ^35^, ^36^ ^]^ The configuration is convenient for producing vector magnetic fields, after calibration, as the current in each coil can be ratioed to produce a desired orientation. Typically, these systems operate at 1 mT and use hundreds of wire turns, with field strengths limited by Ohmic heating and sample distance. Proximal microcoils can cause significant heating, which has adverse implications for sensitive samples, such as in biosensing.^[^ ^37^, ^38^ ^]^ Fields can be significantly increased with the use of superconducting solenoids, but with added costs and constraints.

The constraint of requiring coils at three axes around the sample severely restricts optical or mechanical degrees of freedom. An alternative method is to place and orient a strong neodymium permanent magnet (NdFeB) in the vicinity of the sample. The advantage here is that the small magnet can produce much larger field strengths than the coil. It is less restrictive in its physical footprint, so it can be combined with optical assemblies and cryogenics. The magnets are aligned using linear^[^ ^39^, ^40^, ^41^ ^]^ or rotational translation stages.^[^ ^42^, ^43^ ^]^ The physical limitations of these stages preclude the alignment of certain orientation spin‐based systems.^[^ ^43^ ^]^ Typically, the magnet is positioned once and is aligned along a set diamond crystalline axis because the calibration process is cumbersome. This precludes the use of nanodiamonds where each site may have a random dipole orientation,^[^ ^44^ ^]^ eliminating important applications such as inspecting spatially separated regions or tracking dynamic events in liquid environments. When alignment is not possible, it results in a reduction in sensor performance.^[^ ^45^ ^]^ In general, misaligned magnetic fields have severe implications for spin mixing, flipping, and limited coherence across a range of spin‐based systems.^[^ ^46^, ^47^, ^48^ ^]^

We propose to combine the convenience and control of coils with the small footprint and strength of a permanent magnet in developing a robotically controlled vectorial field alignment system. Our approach has the following advantages: 1) Increased precision and control: The robot manipulates the magnet with a high degree of accuracy, ensuring precise alignment of the generated magnetic field. For our application, this means better than 5° accuracy.^[^ ^30^ ^]^ 2) Fast alignment: By employing a robot to move and position the magnet across optimal trajectories, alignment should be more efficient than manual techniques. 3) Long‐term stability: Employing closed‐loop feedback with sufficient torque against gravity will maintain position securely for extended periods, ensuring stable alignment during experiments. 4) Enhanced reproducibility: A robust algorithm can align and realign the magnet between sample exchanges or across multiple sites of interrogation. The robot consistently produces a given orientation field for different sample geometries. 5) Scalability: the robot has an adaptable routine that is able to suit a wide range of experimental configurations and constraints. One can also easily extend to scenarios where two fields with specific orientations need to be simultaneously applied, for instance, an in‐plane and an out‐of‐plane field.

To position a magnet at a desired location in 3D space with respect to a point of interest, allowing for rotation about two axes to achieve magnetic field orientation, we require a robot with at least five degrees of freedom. The robot must be capable of handling a moderate payload in order to carry enough magnet mass to produce an appreciable field (10 mT) at a distance. It should also be readily available with a well‐developed software interface and be economical to meet the requirements of use outside the robotics community. In the following section, we will evaluate the use of robotics for the described task.

## 2 Results and Discussion

### 2.1 Workspace Analysis

Workspace analysis is essential for robotic control and application, as it evaluates the space the robot can access and manipulate with its end‐effector, constrained by the robot's kinematic configuration. This analysis helps to identify the robot's suitability for specific tasks and environments. Key aspects include the reachable workspace volume (total 3D space the robot's end‐effector can reach), workspace boundaries (limits of reachable space), and dexterity within this workspace (ability to precisely orient the end‐effector).^[^ ^49^ ^]^ In this section, we perform workspace analysis on a magnet carrying robotic arm to evaluate its performance in generating vector magnetic fields.

The robotic arm consists of a set of rigid bodies called links, connected by joints, with each joint driven by a motor actuator. An end‐effector, in this case, a permanent magnet, is attached to the end link. The arm is an open chain robot, with the position and orientation of the end‐effector uniquely determined from the joint positions. The common configuration comprises of six joints, providing six degrees of freedom (DoF). For our experiments, we use a *Niryo NED 2* robot owing to its well‐documented open source stack, and ready availability. The arm has a moderate payload of 300 g, and is thus capable of lifting 40 cm^3^ of NdFeB, which can generate a surface magnetic field of ≈ 800 mT (see Figure S1, Supporting Information). For ease of adoption, we select a cylindrical magnetic source with a radial hole, through which it can be fixed by a screw to the tool shaft. As shown in Figure 1c, the robot is first set up by translating the tool center point (TCP) along the *x*‐axis of its end‐effector, coaxial with the magnetization axis of the magnet (Figure 1a). This translation sets the distance, and hence strength, of the magnet from the point of interrogation.

To create a set vector field, the robot uses the robot operating system (ROS) kinematic processor^[^ ^50^ ^]^ to position its joints in order to compute the desired pose. The robot pose comprises the location and orientation of the TCP relative to a global coordinate frame. Rigid robots possess six state variables (*x*, *y*, *z*, α_*x*_, α_*y*_, α_*z*_), where the latter three coordinates are angles of rotation about the *x*, *y*, and *z*‐axes, respectively. The inverse kinematics problem is to find the joint position given a desired pose. In Table S1, Supporting Information, we give the Denavit–Hartenberg (D–H) representation for the kinematics of this robot. In principle, by fixing *x*, *y*, and *z* at the NV center location, the vector orientation of an applied magnetic field can be modified by varying α_*y*_ and α_*z*_ of the pose. In our scheme, the cylindrical magnet is symmetric about α_*x*_, so this degree of freedom is left unused. In Figure 1c, we simulate in RViz, a visualization software for ROS, that the robot is sufficiently dexterous in positioning its joints to achieve a range of orientations, whereby the magnet is rotated around a stationary point with varying α_*y*_ and α_*z*_. In Figure S2, Supporting Information, we calculate the full workspace volume and dexterity within this volume.

### 2.2 Magnetic Vector Reconstruction

**Figure 2:**
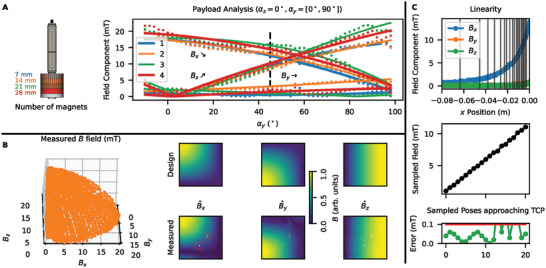
Robot arm generates arbitrary vector magnetic fields. A) A permanent magnet of varying mass is placed in the tool (left panel). A Hall sensor measures the *x*–*z* trajectory of the field produced by the arm (right panel). The trajectory is well‐fitted using a model of the field generated by the cylindrical magnet, noting a 15 ° offset in the *x*–*z* crossing from the expect 45° (shown with dotted line) and a varying non‐zero offset in *y*, with this offset resulting in a non‐linear trend in the field registered with increasing magnetic mass. This initial measurement and model can be used for fine alignment calibration. B) The arm can create a field over the full *x*–*y*–*z* sphere segment (one‐eighth) with 3° accuracy. White pixels in the image plots (circled in red) indicate the few unreachable positions. C) The distance *r* from the end effector to the tool center point (TCP) produces a field strength fall off in *B*_*x*_ proportional to 1/*r* ^3^ (top panel), from which points (shown by vertical lines) can be then sampled (middle panel) to create a linear field response with high accuracy (bottom panel).

The goal of controlling the pose angle is to create a desired magnetic vector field at a given sample location. For experimental verification, we position a 3‐axis Hall sensor at the point of interest in order to measure the field generated from the combined system of the robotic arm and its permanent magnet end‐effector. We set the robot approximately collinear with the sensor axis which is observed using a camera with a zoom lens. In **Figure** **2**a, we see the effect of adding magnets to the structure up to 70% of the payload by setting the robot along an arc trajectory from horizontal to vertical, by rotating the desired pose from α_*y*_ = 0 to α_*y*_ = π/2 with the distance between the sample and magnet surface fixed.

Commonly, in robotics, camera data is processed to extract information on the desired pose.^[^ ^13^ ^]^ Here, the three‐axis Hall sensor provides rich additional vector information, which, coupled with the known dependence of the magnetic field on position, allows the pose to be measured with higher precision than is visually observable. We fit the data with a closed form expression of the magnetic field observed from the cylindrical magnet,^[^ ^51^ ^]^ using the pose variables as fitting parameters. We fit a constant 15° offset in α_*y*_ and observe this in the *x*–*z* crossing point of the sensor and the robot, which for an aligned system would occur at 45° (marked by a dashed line in Figure 2a). Additionally, for this trajectory, we would expect no *B*_*y*_ field to be measured. The non‐zero *B*_*y*_ component is well fit to the varying non‐zero α_*z*_ occurring when each magnet is added. This offset results in a non‐linear relation between the number of magnets added and the observed strength. With an initial calibration trajectory to record this magnetic field information, fine alignment can be achieved either by physically adjusting the robot or by modifying the coordinate frame to correct for the observed misalignment error.

Following this initial trajectory, in Figure 2b, we observe that by scanning through a dictionary of poses, varying only α_*y*_ and α_*z*_, we are able to traverse a set of $B_{\text{abs}}\hat{n}\left(\alpha_{y},\alpha_{z}\right)$ points on the sphere where *B*_abs_ is an approximately constant scalar and $\hat{n}$ is the unit normal vector. In the image plots, we see the measured *B*_*x*_, *B*_*y*_, and *B*_*z*_ over each pose α_*y*_, α_*z*_ compared to the designed field. There is a small percentage of white pixels representing poses within the workspace that were unachievable by the kinematic processor. The robot scans in a meander, alternating +*z*, −*z*, and artefacts of this are seen through scan lines in the measured data. Overall, we measure high angular accuracy with a mean error of 2.9° and mode error of 2.3° and confirm that the robotic arm is able to produce desired field orientations with a high accuracy. In measuring the field components against the designed pose, this accuracy captures uncertainty in the motor actuators, the corresponding pose of the robot, the analytical model of the field generated, and the field measured by the Hall sensor. This measurement confirms alignment is achievable with this system over all vector orientations below the target 5° threshold.

### 2.3 Field Amplitude Control

For a set vector orientation and magnet mass, some ODMR applications require tuning of the magnetic field amplitude, for instance so that the spin resonance frequency matches a microwave resonator.^[^ ^52^, ^53^ ^]^ The field amplitude can be controlled by tuning the distance between the magnet and the sample position. However, the magnetic field fall‐off with *r* distance is highly non‐linear, characterized by the Biot–Savart 1/*r* ^3^ relation. In addition, the robotic arm performs non‐linear displacement, requiring dual movement of two rotational joints per linear step of the end effector.

We observe in Figure 2c that the displacement of the magnet away from the Hall sensor is sufficiently linear to produce a 1/*x* ^3^ response in *B*_*x*_. Because the magnetic field generated by the permanent magnet is large, it can be positioned sufficiently far away from the sensor so that the 10 mm 1/*r* ^3^ trajectory can be subsampled within the 0.5 mm resolution of the robot (lines shown in the top panel) to create a desired response *B*(*r*). In the middle panel, we observe that we can create a linear field response between 0 and 10 mT through this method. In the bottom panel, we observe the error in this sampling technique is typically lower than 0.1 mT. However, as expected, this error increases for close distances to the sensor as the available resolution to subsample the 1/*r* ^3^ field diminishes.

### 2.4 Collision‐Free Motion Planning

With operation validated in an unconstrained environment, we move to navigating the robot around complicated lab infrastructure. By evaluating intersections with its environment, the robot is able to compute collisions in the ROS simulation using LBKPiece from the Open Motion Planning Library to traverse a tree of possible trajectories to achieve a given pose goal.^[^ ^54^ ^]^

**Figure 3:**
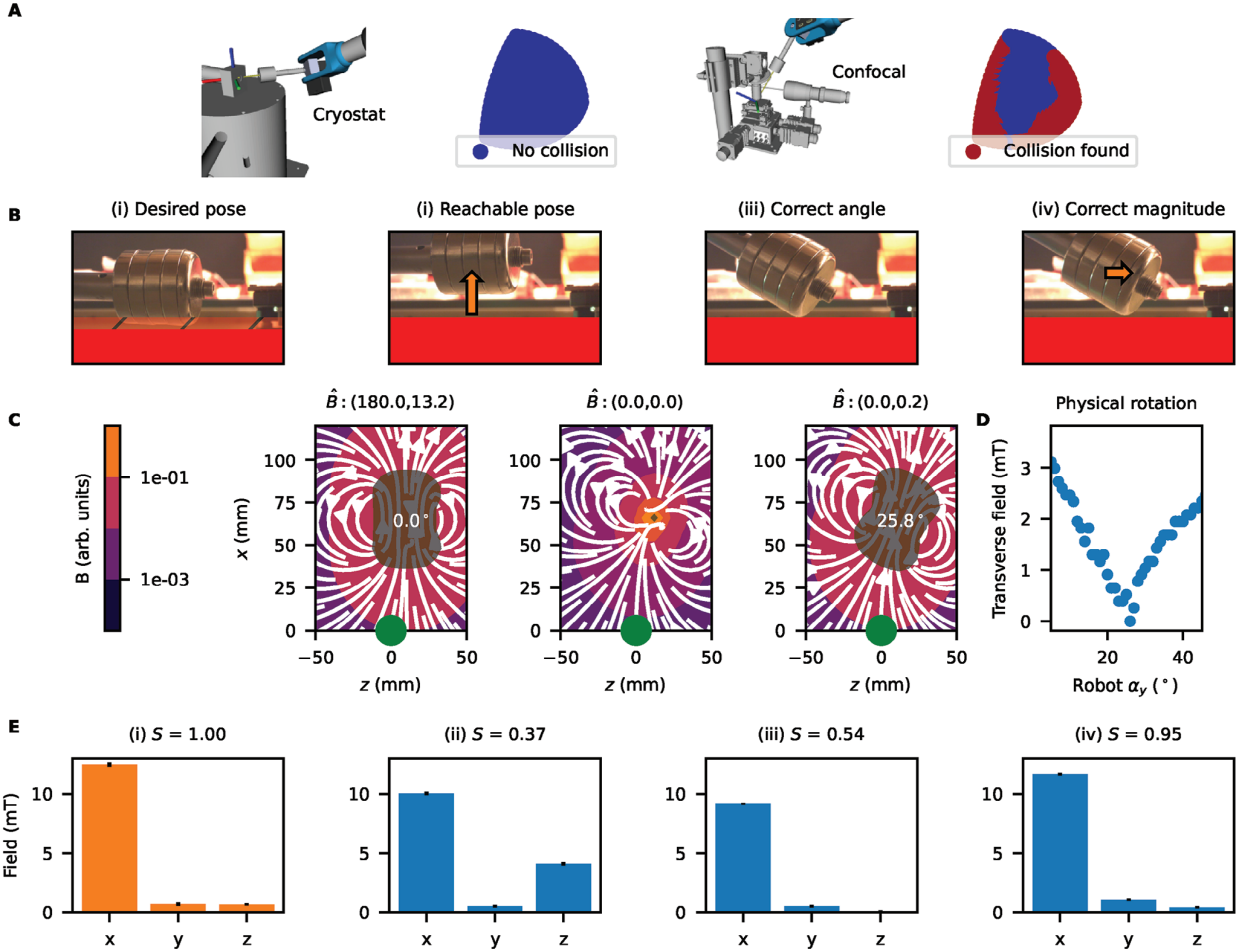
Motion planning in experimental settings. A) We model two experimental settings: a cryostat with optical access and a confocal microscope. Plotted are the simulated collisions with the robot (in red) and avoided collisions (in blue) over the fixed position varied (α_*y*_, α_*z*_) poses. For the confocal, we observe a limited reachable workspace subset. B) We develop an algorithm to replace these unreachable poses with collision‐free poses. The procedure follows: i) a desired field vector is measured in a forbidden position; ii) displacement puts the magnet in an allowed position; iii) angular orientation sets the correct field vector; and iv) a further displacement corrects the field magnitude. C) Using a dipole source to calculate the rotation, we can deterministically rotate the magnet and recover the $\hat{B}$ vector (title bar) at the observer (green dot). D) The designed rotation matches experimentally in minimizing the transverse field at 26°. E) Measuring with the 3D Hall sensor at each stage following panels in (B), the final vector well matches the initial vector (quantified by the similarity function *S* defined in the text).

We take two experimental setups in our lab, a cryostat with an optical window and a scanning stage confocal microscope (see **Figure** **3**a), and add their spatial meshes in simulation to the robot environment. For these complex geometries, it would not be possible to position three‐axis Helmholtz coils for magnetic field alignment due to the competing requirements for optical access to the sample and the need to move the sample in 3D. Single microcoils or a permanent magnet mounted on a stage would have limitations in terms of achievable proximity to the sample.

With the available kinematics of the 6 DoF robotic arm, the position of the magnet with respect to the sample is far less constrained. In Figure 3a, using LBKPiece, we simulate that a chosen subset of poses can be traversed with access to the top and back sides of the cryostat, oriented around the TCP located at the sample mount, generating a −*B*_*x*_, +*B*_*y*_, −*B*_*z*_ sphere segment (one‐eighth) without collisions. However, we observe that for the scanning stage confocal microscope, only a subset of any sphere segment is achievable. Poses that cannot be accessed without collision are shown with red dots and form a significant part of the subset. From this simulation, it is evident that there would have limited success of the robot in the magnetization‐axis‐aligned configuration described in Figure 1c.

### 2.5 Designing Collision‐Free Field Vectors

An important consideration at this point is that the set of poses in this configuration only make a small subset of the possible joint configurations of the robot and therefore possible magnetic field vectors. By moving the TCP defined in Figure 1c from the NV center to the magnet, we give free control over its orientation and position, with access to the fringing fields of the magnetic source. Our hypothesis is that there exists a set of collision‐free poses that would produce a full set of magnetic vectors. This idea makes use of the magnetic inverse problem in field sensing: even if a pose cannot be reached, the desired field can be obtained because there is a non‐unique mapping between the field and the pose.^[^ ^55^ ^]^ Our algorithm is laid out in Figure 2b. First, the unreachable set of poses in the constrained environment is found. For each such pose (Figure 2b(i)), the TCP is translated along *x* to the magnet center and the magnet is then linearly translated in either *y* or *z* to a new reachable pose (Figure 2b(ii)). Next, the new pose is rotated in α_*y*_ or α_*z*_ to obtain the same vector field orientation as the original pose (Figure 2b(iii)). Finally, the magnet is translated along *x* to recover the original magnitude (Figure 2b(iv)).

To calculate the vector rotation in Figure 2b(iii), we can approximate the magnet with a dipole, for which the inverse magnetostatic expression is known.^[^ ^55^ ^]^ For a powerful permanent magnet, the arm can be withdrawn at sufficient distances so that this dipole approximation becomes valid. The orientation of a unit dipole $\vec{m}$ at a vector $\vec{r}$ to create a field at the sensor location $\vec{B}$ is given by

(2) $$\begin{array}{c} \vec{m}=\frac{6\pi }{\mu_{0}}\left(\vec{B}\cdot \vec{r}\right)\left|\vec{r}\right|\vec{r}-\frac{4\pi }{\mu_{0}}{\left|\vec{r}\right|}^{3}\vec{B}, \end{array}$$

where μ_0_ is the vacuum permeability.

In Figure 3c, we model the *z* displaced magnet and find that the field observed at the sample location (green dot) has a significantly modified orientation (first panel). We can use Equation (2) to calculate the dipole orientation $\vec{m}$ to find $\vec{B}$ (middle panel). We then rotate the magnet to be coaxial with the calculated dipole orientation and recover the desired field vector $\vec{B}$ with high accuracy, minimizing *B*_*y*_ and *B*_*z*_ (last panel). In Figure 3d, we show that in the physical experiment, the off‐axial field component is indeed minimized when set to the calculated 26° angle, and that this algorithm succeeds within the pose resolution limit of the robot.

As well as correcting orientation, for some applications it is important to maintain the field amplitude. This final step in Figure 3b(iv) is achieved by using the known 1/*r* ^3^ Biot–Savart relation of Figure 2c to scale the amplitude, translating the magnet in *x*. To capture both the orientation and amplitude, we define a Gaussian kernel similarity function between the target field vector *B*_1_ and the replacement vector *B*_2_, as this is well bounded between 0 (least similar) and 1 (most similar):

(3) $$\begin{array}{c} S=\exp\frac{\left|\right|B_{2}-B_{1}{\left|\right|}^{2}}{2d^{2}} \end{array}$$

with *d* = 3. We experimentally implement each step of the algorithm in Figure 3e and see that the final vector achieves a high similarity to the target vector with *S* = 0.95. This can also be seen by comparing the histograms of the measured field components in Figure 3e(i,iv). Here, the final amplitude correcting step maintains the desired field orientation. We have evidenced that by using this algorithm, it is possible to systematically replace unreachable poses with reachable poses with the same field vector. The full dexterity offered by the robotic links combined with the inverse problem of magnetostatics make this system a powerful tool for setting arbitrary‐strength magnetic field vectors in highly constrained environments. We note that this model works in standard laboratories without the presence of ferromagnets. Field distortions in the presence of iron or nickel objects should be calculated if present.

### 2.6 Experimental Setting of an NV Center Confocal Microscope

**Figure 4:**
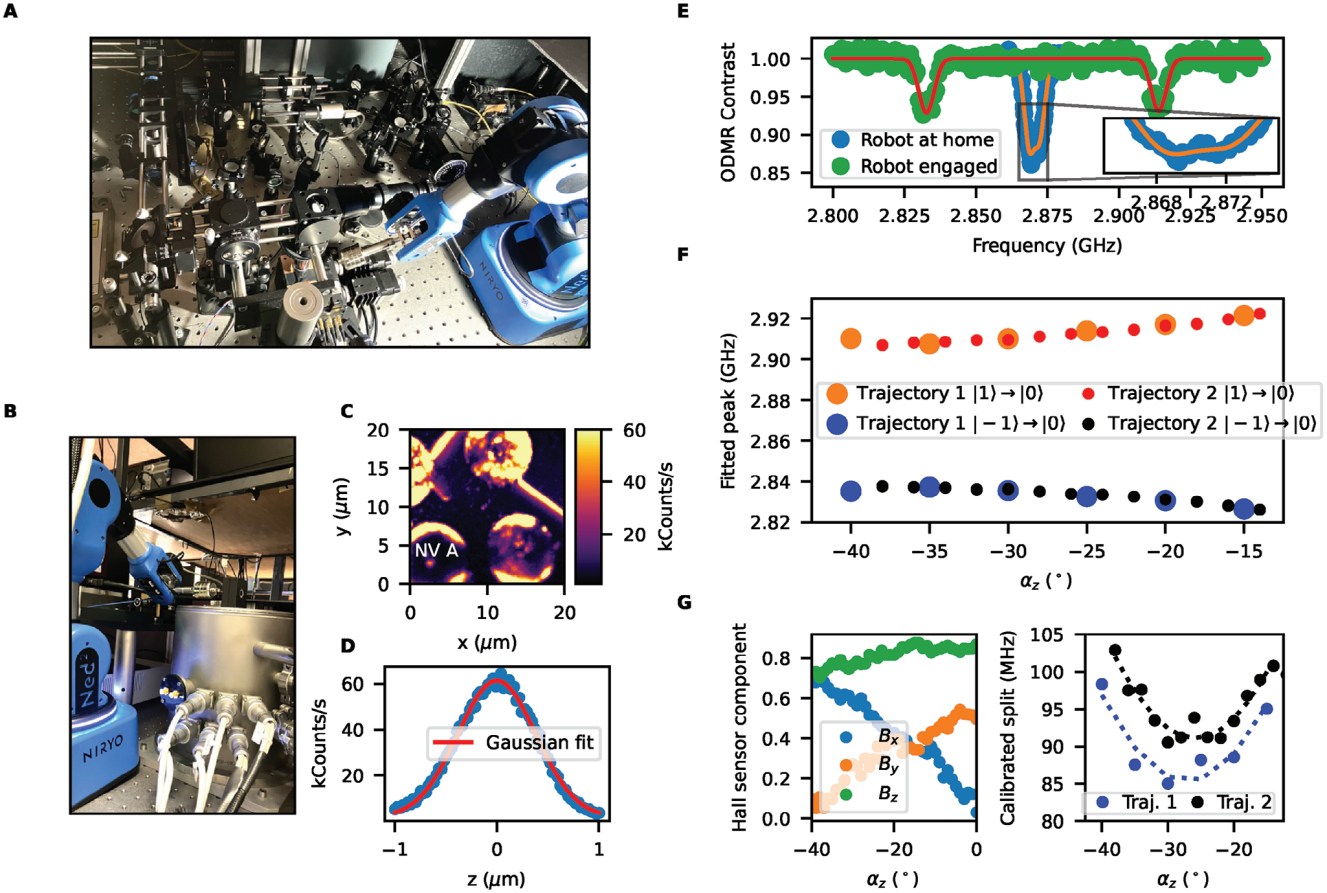
Robot‐assisted magnetometry: A) Image of confocal microscope setup showing robotic arm in position. B) Optically accessed cryostat with robotic arm in position. C) Confocal image of NV center located in diamond lens (mounted in setup shown in A). D) Photoluminescence scan in *z* of above. E) Optically detected magnetic resonance (ODMR) showing zero‐field magnetic field splitting of associated spin in blue when the robot arm is ≈10 cm from the sensor and strong 100 MHz splitting in green when the robot arm is proximal to the spin sensor. F) Fitted peak resonance for robot trajectory in 5° increments (large circles) and 2° increments (small circles), indicating movement is stable and repeatable. G) Hall sensor data shows the *B*_*x*_ and *B*_*y*_ crossover in trajectory. Normalizing splitting between resonances by *B* magnitude reveals σ_*z*_ dependence in Trajectory 1 (black) and Trajectory 2 (blue), indicative of angular alignment.

Following validation with the Hall sensor, we now move to a full experimental setting in order to evaluate the performance of the robot for aligning a spin based quantum sensor. We see in **Figure** **4**a,b that this requires navigating a highly complex environment with many sensitive optical and mechanical instruments.

In the technique described in the previous section, the collision‐free positions found in simulation can be downloaded to the physical robot. This requires a fine alignment between the simulation and the real world performance, which could be achieved using the approach described in Figure 2a. In a highly constrained environment, we find sensor‐driven fine angular alignment is difficult to achieve as the initial calibration trajectories contain poses that cannot be verified as collision‐free until this alignment has succeeded. In a future iteration, additional sensor data could enrich this information using machine vision or ultrasound to map the collision surroundings.

A fast and pragmatic approach in an experimental setting is to *teach* the robot a set of collision‐free poses. We do this by switching the torque on each motor off momentarily, allowing the joints to move freely. Now, the operator can grasp the magnet and guide the robotic arm to a desired location within the geometry, avoiding collision. Whilst doing this, the user can monitor the magnetic field produced at the sample location with the Hall sensor. When a desired field is registered, the torque and closed loop feedback can be switched on, locking the magnet in its set position. The corresponding coordinates (either the pose or the joint angles) can be registered along with the measured magnetic field. Through this method, the user can hunt and find locations where, for instance, *z* is the dominant field component. These poses can be used to gather information to calibrate the source to its experimental surroundings. We can both compare the taught poses with the simulation to calculate collisions and with the dipole field model, we can locally modify the taught position to achieve the desired magnetic field vectors.

As a proof‐of‐principle experiment, we teach the robot a trajectory across the confocal microscope shown in Figure 4a. We can then demonstrate that traversing this trajectory repeatably affects the NV center spin based sensor. In Figure 4c, we use the confocal microscope to locate a collection of milled solid immersion lenses in a polycrystalline diamond sample,^[^ ^56^ ^]^ observing a bright NV center in the central lens (Figure 4d). With the robot arm at its home position, 10 cm away from the sample, we perform ODMR on this NV center (see Experimental Section for experimental details). As defined in Equation (1), we observe a small 3.7030 MHz splitting due to an intrinsic Π = 1.8515 MHz, about the central *D* = 2.8704 GHz, shown in Figure 4e. In moving the TCP near the NV center site, with the robot end effector in the vicinity of the experimental setup, we observe a large 100 MHz splitting in the ODMR spectra (green plot) from the presence of the permanent magnet. Typically, the NV center must be optically aligned to the photon detector within 500 nm using a piezo stage and the ability to engage and disengage the robot whilst performing ODMR indicates it is suitable to use in this highly sensitive experiment.

### 2.7 Robotic Vectorial Field Alignment of a Single Spin Sensor

In Figure 4f, we repeat the ODMR for a set of robot poses along a collision‐free trajectory with α_*y*_ = 20 ° at 5 ° increments in α_*z*_ (Trajectory 1). Each data point was averaged over 5 min. The peak resonances show a smooth increase in splitting from *D* as the magnet is moved in front of the objective. The same path is traversed at 2° increments in α_*z*_ (Trajectory 2) and resonance data shows the same splitting trend, indicating that the trajectory‐induced splitting is reproducible. The fitted resonances can be used to extract the polar angle between the NV axis and the magnetic field of interrogation, from the zero‐field parameters *D* and Π (see Experimental Section for details).^[^ ^57^ ^]^ This gives a value of 61.0 ± 0.3° for Trajectory 1 and 61.8 ± 0.2° for Trajectory 2.^[^ ^57^ ^]^ This small polar angle change over the 25 ° range in α_*z*_ indicates that the NV center is near aligned with the world coordinate *z*‐axis.

In Figure 4g, by replacing the diamond sample with the Hall sensor and focusing the objective at the center of the sensor area, we can map the field components generated by the trajectory. This alignment step co‐locates the relative pose between the sensor and the robot as described in Figure S3, Supporting Information. We see that the *B*_*x*_ and *B*_*y*_ components cross, as expected for varying α_*z*_. We find the Hall field amplitude |*B*| in excellent agreement with the ODMR sensed field amplitude (see Figure S3, Supporting Information). For the trajectory chosen, the robot TCP is not yet fully aligned with the NV or Hall sensor area. We see that in contrast to Figure 2b, the trajectory does not conserve |*B*|, with a large increasing *B*_*z*_ component, and this is the main contribution to the increased splitting between the two resonances in Figure 4f.

Using the amplitude data, we can normalize each splitting to isolate the field component ratios (see Experimental Section). In Figure 4g, this normalization results in the appearance of a dip from both trajectories, with the phase and contrast of the dip giving an estimation of the NV's orientation. The low contrast, non‐zero minima in α_*z*_ indicates the NV center is near aligned with the world coordinate *z*‐axis as previously discussed. We find that both datasets can be well fitted with the characteristic equation^[^ ^34^ ^]^ to find α_*z*_ = 64.1 ± 0.4°, α_*y*_ = 97.6 ± 0.7° for Trajectory 1 and α_*z*_ = 62.9 ± 0.8°, α_*y*_ = 97.9 ± 1.4° for Trajectory 2. In this, we have shown that vectorial field alignment can be achieved with robotic trajectories of as little as six steps. This is especially useful for ODMR, where collecting each spectra takes on the order of minutes.

## 3 Conclusion

Our results show that an industrially designed robotic arm can be adapted to operate around sensitive optomechanical samples and setups. The presented modality produces stable and controllable magnetic fields that are capable of manipulating and aligning a single solid state quantum spin sensor. This is an important step in the use of robotics to replace axial stages and bulky field coils for experimental physics and in developing quantum technologies, where we have evidenced the benefit of the innate flexibility and configurability of robotic arms in highly constrained environments.

The next step in this work is to generate on‐demand magnetic fields using a sophisticated algorithm that maps the traversable space given geometrical parameters, making use of the collision‐free techniques described. With this, a set of control points can be found, considering application‐specific criteria such as the field magnitude, linearization, or the time taken to move between points.

Robotics, unlike solenoid coils, produce minimal local heat. This makes them suited for sensitive samples, and algorithms could be designed for tracking quantum sensors in motion under cell uptake, a difficult task where the spin sensor orientation changes over time.^[^ ^58^, ^59^ ^]^ For further flexibility, the cylindrical magnet could be replaced with a rectangular magnet fixed perpendicular to its magnetic axis, with the unused roll degree of freedom in the robotic wrist providing rapid field orientation.

Beyond an off‐the‐shelf design, an application‐specific robot could further maximize efficiency, precision, and control. This could have a larger payload whilst having a smaller form factor, for instance. We can extend this to the use of multiple robots to generate gradient magnetic fields. Without our approach, we find a 99% field uniformity over millimeter volumes (see Figure S1, Supporting Information). However, this is significantly less than Helmholtz coils, which maintain this uniformity over their central volume (> cm^3^). To combat this, multiple robots could be combined to increase uniformity over large areas. As well as a range of solid‐state sensors, the alignment of atoms and ions in cold and vacuum environments can be explored with these form factors. A probe‐like flux concentrator appended to the end‐effector could achieve higher field strengths at distant locations, although the presence of this ferromagnet would have to be robustly modeled.^[^ ^60^ ^]^

In addition, the robot‐driven orientation presented can be extended to aligning quantum objects with a range of parameters, including electric and light fields. Here, the end effector would be an electrode, or in optics, a laser or mirror surface. Following this proof‐of‐principle work, the adaptability of robots in combination with sophisticated software could provide ruggedness for alignment in demanding real‐world environments where quantum technologies are emerging, such as point to point quantum key distribution (QKD)^[^ ^61^ ^]^ and quantum range finding.^[^ ^62^ ^]^

## 4 Experimental Section

### Magnetic Field Modeling

Magnetic field calculations in this work were performed using the closed form expressions presented in the Magpylib package.^[^ ^51^ ^]^ The hollow cylindrical magnet was modeled by subtracting an inner cylindrical magnetic source of opposite magnetization from the outer cylindrical magnet source.

### Robotic Modeling

The Niryo NED 2 robot geometry was specified in the unified robot description format (URDF). Here, the end‐effector geometry file specified in the URDF was replaced with the geometry of the magnet tool. For the collision‐free motion path finding, the experimental setups were modeled in FreeCad, and the geometry file of the robot base was replaced. The robot was simulated in an ROS environment and controlled using the Python wrapper PyNiryo2.

### Experimental Setup

The magnetic field measurements in this work were made using the Infineon TLE493D‐P2B6MS2GO 3D magnetic sensor fitted on a compact platform mount or in the described confocal microscope. For the ODMR measurements, the NV center was excited by a CW 532 nm laser (gem 532; Laser Quantum). A confocal microscope was used to image the collected count rate. Using a 0.9 NA microscope objective, the excitation beam was highly focused on the sample, producing a nearly diffraction‐limited spot (< 1 μm diameter). The NV center PL was collected through the same lens and separated from the excitation path by the use of a dichroic mirror before detection by single photon avalanche diodes (SPADs) (SPCM‐AQRH‐12‐FC; Excelitas). By scanning the position of the sample, a map of the detected count rate was generated, from which the position of the NV center and its maximum count rate could be found. ODMR was performed under CW excitation using a Rohde and Schwarz SMB100A microwave source driving a custom loop antenna PCB on which the sample was mounted.

### Spin Sensor Modeling

The spin transitions presented in Figure 1b were calculated by solving the Hamiltonian eigenstates in QuTiP.^[^ ^63^ ^]^ Other calculations used the NV spin energies characteristic polynomial presented in Balasubramanian et al., whereas the polar angle θ between the field and the NV center was found using the solution given in that work.^[^ ^57^ ^]^ The polynomial could be solved and least‐squared fitted to the experimental data to find the NV orientation $\left(\alpha_{y}^{\mathrm{NV}},\alpha_{z}^{\mathrm{NV}}\right)$,^[^ ^34^ ^]^

(4) $$\begin{array}{c} \begin{array}{cc} x^{3}-\left(\frac{D^{2}}{3}+\Pi^{2}+\beta^{2}\right)x-\frac{\beta^{2}}{2}D\cos2\gamma & -\frac{D}{6}\left(4\Pi^{2}+\beta^{2}\right)\\ & +\frac{2D^{3}}{27}=0 \end{array} \end{array}$$

where $\gamma =\arccos(|\cos\left(\alpha_{z}^{B}-\alpha_{z}^{\mathrm{NV}}\right)\cos\left(\alpha_{y}^{B}-\alpha_{y}^{\mathrm{NV}}\right)|)$ with a known $\alpha_{y}^{B}$ and $\alpha_{z}^{B}$ set by the robot, *D* and Π fitted from the zero‐field data, and β = γ_e_|*B*| where γ_e_ is the gyromagnetic ratio and |*B*| is the external magnetic field amplitude. In the trajectories presented, and the separation between resonances ν(*i*), |*B*| was not conserved. For this fit, a constant |*B*| must be obtained, so it must first be normalized using the field magnitude data. For this, the higher resolution Hall data was sub‐sampled to reduce noise (see Figure S3, Supporting Information) and obtain normalized splittings ν_*n*_(*i*) for each measurement point *i* where:

(5) $$\begin{array}{c} \nu_{n}\left(i\right)=\frac{\nu (i)}{|B_{\text{Hall}}\left(i\right)|}\max\left|B_{\text{Hall}}\right| \end{array}$$

and leave the non‐physical *B* in the characteristic equation as the third free parameter in the fit to this data.

### Statistical Analysis

Raw Hall data and confocal photoluminescence data were presented as collected. ODMR data was processed using Python. Spectra was two‐point resampled by summing adjacent bins in Numpy to aid fitting. LMFit was used to fit Gaussian centroids. LMFit was then used to sinusoid‐fit the centroids. For this, Numpy was used to normalize the trajectory using the Hall data as described in the previous section. All data was visualized using Matplotlib in Python.

## Conflict of Interest

The authors declare no conflict of interest.

## Supporting information

Supporting Information

## Data Availability

The data that support the findings of this study are available from the corresponding author upon reasonable request.

## References

[1] X. Qiang , X. Zhou , J. Wang , C. M. Wilkes , T. Loke , S. O'Gara , L. Kling , G. D. Marshall , R. Santagati , T. C. Ralph , J. B. Wang , J. L. O'Brien , M. G. Thompson , J. C. F. Matthews , Nat. Photonics 2018, 12, 534.

[2] H.‐S. Zhong , Y.‐H. Deng , J. Qin , H. Wang , M.‐C. Chen , L.‐C. Peng , Y.‐H. Luo , D. Wu , S.‐Q. Gong , H. Su , Y. Hu , P. Hu , X.‐Y. Yang , W.‐J. Zhang , H. Li , Y. Li , X. Jiang , L. Gan , G. Yang , L. You , Z. Wang , L. Li , N. L. Liu , J. J. Renema , C.‐Y. Lu , J.‐W. Pan , Phys. Rev. Lett. 2021, 127, 180502. 34767431 10.1103/PhysRevLett.127.180502

[3] V. V. Soshenko , S. V. Bolshedvorskii , O. Rubinas , V. N. Sorokin , A. N. Smolyaninov , V. V. Vorobyov , A. V. Akimov , Phys. Rev. Lett. 2021, 126, 197702. 34047600 10.1103/PhysRevLett.126.197702

[4] D. Kielpinski , C. Monroe , D. J. Wineland , Nature 2002, 417, 709. 12066177 10.1038/nature00784

[5] S. Krinner , S. Storz , P. Kurpiers , P. Magnard , J. Heinsoo , R. Keller , J. Luetolf , C. Eichler , A. Wallraff , EPJ Quantum Technol. 2019, 6, 2.

[6] G. Kurizki , P. Bertet , Y. Kubo , K. Mølmer , D. Petrosyan , P. Rabl , J. Schmiedmayer , Proc. Natl. Acad. Sci. U. S. A. 2015, 112, 3866. 25737558 10.1073/pnas.1419326112 PMC4386362

[7] A. Clerk , K. Lehnert , P. Bertet , J. Petta , Y. Nakamura , Nat. Phys. 2020, 16, 257.

[8] E. Albertinale , L. Balembois , E. Billaud , V. Ranjan , D. Flanigan , T. Schenkel , D. Estève , D. Vion , P. Bertet , E. Flurin , Nature 2021, 600, 434. 34912088 10.1038/s41586-021-04076-z

[9] S. Hermans , M. Pompili , H. Beukers , S. Baier , J. Borregaard , R. Hanson , Nature 2022, 605, 663. 35614248 10.1038/s41586-022-04697-y PMC9132773

[10] S. J. Whiteley , G. Wolfowicz , C. P. Anderson , A. Bourassa , H. Ma , M. Ye , G. Koolstra , K. J. Satzinger , M. V. Holt , F. J. Heremans , A. N. Cleland , D. I. Schuster , G. Galli , D, D. Awschalom , Nat. Phys. 2019, 15, 490.

[11] K. C. Balram , K. Srinivasan , Adv. Quantum Technol. 2022, 5, 2100095.

[12] M. Akhtar , F. Bonus , F. Lebrun‐Gallagher , N. Johnson , M. Siegele‐Brown , S. Hong , S. Hile , S. Kulmiya , S. Weidt , W. Hensinger , Nat. Commun. 2023, 14, 531. 36754957 10.1038/s41467-022-35285-3 PMC9908934

[13] D. Zhang , A. Barbot , F. Seichepine , F. P.‐W. Lo , W. Bai , G.‐Z. Yang , B. Lo , Commun. Phys. 2022, 5, 80.

[14] J. Cline , M. Vaughan , W. J. Waltz , I. M. Wong , in AIAA SCITECH 2022 Forum , American Institute of Aeronautics and Astronautics, Reston, VA 2022, pp. 0625.

[15] J. M. Granda , L. Donina , V. Dragone , D.‐L. Long , L. Cronin , Nature 2018, 559, 377. 30022133 10.1038/s41586-018-0307-8 PMC6223543

[16] J. Durrer , P. Agrawal , A. Ozgul , S. C. Neuhauss , N. Nama , D. Ahmed , Nat. Commun. 2022, 13, 6370. 36289227 10.1038/s41467-022-34167-y PMC9605990

[17] C. L. Degen , F. Reinhard , P. Cappellaro , Rev. Mod. Phys. 2017, 89, 035002.

[18] E. Van Oort , N. Manson , M. Glasbeek , J. Phys. C: Solid State Phys. 1988, 21, 4385.

[19] L. Rondin , J.‐P. Tetienne , T. Hingant , J.‐F. Roch , P. Maletinsky , V. Jacques , Rep. Prog. Phys. 2014, 77, 056503. 24801494 10.1088/0034-4885/77/5/056503

[20] F. Casola , T. Van Der Sar , A. Yacoby , Nat. Rev. Mater. 2018, 3, 17088.

[21] A. Ajoy , U. Bissbort , M. D. Lukin , R. L. Walsworth , P. Cappellaro , Phys. Rev. X 2015, 5, 011001.

[22] A. Ermakova , G. Pramanik , J.‐M. Cai , G. Algara‐Siller , U. Kaiser , T. Weil , Y.‐K. Tzeng , H.‐C. Chang , L. McGuinness , M. B. Plenio , B. Naydenov , F. Jelezko , Nano Lett. 2013, 13, 3305. 23738579 10.1021/nl4015233

[23] I. Lovchinsky , A. Sushkov , E. Urbach , N. P. de Leon , S. Choi , K. De Greve , R. Evans , R. Gertner , E. Bersin , C. Müller , L. McGuinness , F. Jelezko , R. L. Walsworth , H. Park , M. D. Lukin , Science 2016, 351, 836. 26847544 10.1126/science.aad8022

[24] L. P. McGuinness , Y. Yan , A. Stacey , D. A. Simpson , L. T. Hall , D. Maclaurin , S. Prawer , P. Mulvaney , J. Wrachtrup , F. Caruso , R. E. Scholten , L. C. L. Hollenberg , Nat. Nanotechnol. 2011, 6, 358. 21552253 10.1038/nnano.2011.64

[25] L. Nie , A. C. Nusantara , V. G. Damle , M. V. Baranov , M. Chipaux , C. Reyes‐San‐Martin , T. Hamoh , C. P. Epperla , M. Guricova , P. Cigler , G. Van Den Bogaart , R. Schirhag , Nano Lett. 2021, 22, 1818. 34929080 10.1021/acs.nanolett.1c03021 PMC8880378

[26] M. Alkahtani , J. Lang , B. Naydenov , F. Jelezko , P. Hemmer , ACS Photonics 2019, 6, 1266.

[27] T. Mittiga , S. Hsieh , C. Zu , B. Kobrin , F. Machado , P. Bhattacharyya , N. Rui , A. Jarmola , S. Choi , D. Budker , N. Y. Yao , Phys. Rev. Lett. 2018, 121, 246402. 30608732 10.1103/PhysRevLett.121.246402

[28] F. Dolde , H. Fedder , M. W. Doherty , T. Nöbauer , F. Rempp , G. Balasubramanian , T. Wolf , F. Reinhard , L. C. Hollenberg , F. Jelezko , J. Wrachtrup , Nat. Phys. 2011, 7, 459.

[29] J. Tetienne , L. Rondin , P. Spinicelli , M. Chipaux , T. Debuisschert , J. Roch , V. Jacques , New J. Phys. 2012, 14, 103033.

[30] P. L. Stanwix , L. M. Pham , J. R. Maze , D. Le Sage , T. K. Yeung , P. Cappellaro , P. R. Hemmer , A. Yacoby , M. D. Lukin , R. L. Walsworth , Phys. Rev. B 2010, 82, 201201.

[31] J. Maze , J. Taylor , M. Lukin , Phys. Rev. B 2008, 78, 094303.

[32] M. Geiselmann , M. L. Juan , J. Renger , J. M. Say , L. J. Brown , F. J. G. De Abajo , F. Koppens , R. Quidant , Nat. Nanotechnol. 2013, 8, 175. 23396312 10.1038/nnano.2012.259

[33] J. M. Schloss , J. F. Barry , M. J. Turner , R. L. Walsworth , Phys. Rev. Appl. 2018, 10, 034044.

[34] K. Fukushige , H. Kawaguchi , K. Shimazaki , T. Tashima , H. Takashima , S. Takeuchi , Appl. Phys. Lett. 2020, 116, 264002.

[35] L. Stefan , A. K. Tan , B. Vindolet , M. Högen , D. Thian , H. K. Tan , L. Rondin , H. S. Knowles , J.‐F. Roch , A. Soumyanarayanan , M. Atatüre , Phys. Rev. Appl. 2021, 16, 014054.

[36] S. Knauer , J. P. Hadden , J. G. Rarity , npj Quantum Inf. 2020, 6, 50.

[37] H. Zhang , K. Arai , C. Belthangady , J.‐C. Jaskula , R. L. Walsworth , NPJ Quantum Inf. 2017, 3, 31.

[38] G. Petrini , E. Moreva , E. Bernardi , P. Traina , G. Tomagra , V. Carabelli , I. P. Degiovanni , M. Genovese , Adv. Quantum Technol. 2020, 3, 2000066.

[39] T. Weggler , C. Ganslmayer , F. Frank , T. Eilert , F. Jelezko , J. Michaelis , Nano Lett. 2020, 20, 2980. 32182080 10.1021/acs.nanolett.9b04725

[40] A. Stark , Ph.D. Thesis , Technical University of Denmark, 2017.

[41] A. Cooper , W. K. C. Sun , J.‐C. Jaskula , P. Cappellaro , Phys. Rev. Lett. 2020, 124, 083602. 32167360 10.1103/PhysRevLett.124.083602

[42] J. Holzgrafe , Q. Gu , J. Beitner , D. M. Kara , H. S. Knowles , M. Atatüre , Phys. Rev. Appl. 2020, 13, 044004.

[43] B. Wood , G. Stimpson , J. March , Y. Lekhai , C. Stephen , B. Green , A. Frangeskou , L. Ginés , S. Mandal , O. Williams , G. W. Morley , Phys. Rev. B 2022, 105, 205401.

[44] N. Aslam , H. Zhou , E. K. Urbach , M. J. Turner , R. L. Walsworth , M. D. Lukin , H. Park , Nat. Rev. Phys. 2023, 5, 157. 36776813 10.1038/s42254-023-00558-3 PMC9896461

[45] T. Jung , J. Görlitz , B. Kambs , C. Pauly , N. Raatz , R. Nelz , E. Neu , A. M. Edmonds , M. Markham , F. Mücklich , J. Meijer , C. Becher , APL Photonics 2019, 4, 12.

[46] C. Nguyen , D. Sukachev , M. Bhaskar , B. Machielse , D. Levonian , E. Knall , P. Stroganov , C. Chia , M. Burek , R. Riedinger , H. Park , M. Loncar , M. D. Lukin , Phys. Rev. B 2019, 100, 165428. 10.1103/PhysRevLett.123.183602 31763904

[47] P. Klimov , A. Falk , B. Buckley , D. Awschalom , Phys. Rev. Lett. 2014, 112, 087601. 10.1103/PhysRevLett.112.187601 24856721

[48] M. Widmann , S.‐Y. Lee , T. Rendler , N. T. Son , H. Fedder , S. Paik , L.‐P. Yang , N. Zhao , S. Yang , I. Booker , A. Denisenko , M. Jamali , S. A. Momenzadeh , I. Gerhardt , T. Ohshima , A. Gali , E. Janzén , J. Wrachtrup , Nat. Mater. 2015, 14, 164. 25437256 10.1038/nmat4145

[49] D. Zhang , F. Cursi , G.‐Z. Yang , IEEE Rob. Autom. Lett. 2019, 4, 3836.

[50] M. Quigley , K. Conley , B. Gerkey , J. Faust , T. Foote , J. Leibs , R. Wheeler , A. Y. Ng , ICRA Workshop Open Source Software 2009, 3, 5.

[51] M. Ortner , L. G. Coliado Bandeira , SoftwareX 2020, 11, 100466.

[52] A. Angerer , K. Streltsov , T. Astner , S. Putz , H. Sumiya , S. Onoda , J. Isoya , W. J. Munro , K. Nemoto , J. Schmiedmayer , J. Majer , Nat. Phys. 2018, 14, 1168.

[53] S. Putz , D. O. Krimer , R. Amsuess , A. Valookaran , T. Noebauer , J. Schmiedmayer , S. Rotter , J. Majer , Nat. Phys. 2014, 10, 720.

[54] I. A. Sucan , M. Moll , L. E. Kavraki , IEEE Rob. Autom. Mag. 2012, 19, 72.

[55] E. A. Lima , A. Irimia , J. P. Wikswo , in The SQUID Handbook, Vol. II: Applications of SQUIDs and SQUID Systems (Eds: J. Clarke , A. I. Braginski ), Wiley, New York 2006, Ch. 10, pp. 139–267.

[56] J. Hadden , J. Harrison , A. C. Stanley‐Clarke , L. Marseglia , Y.‐L. Ho , B. Patton , J. L. O'Brien , J. Rarity , Appl. Phys. Lett. 2010, 97, 241901.

[57] G. Balasubramanian , I. Chan , R. Kolesov , M. Al‐Hmoud , J. Tisler , C. Shin , C. Kim , A. Wojcik , P. R. Hemmer , A. Krueger , T. Hanke , A. Leitenstorfer , R. Bratschitsch , F. Jelezko , J. Wrachtrup , Nature 2008, 455, 648. 18833276 10.1038/nature07278

[58] D. Le Sage , K. Arai , D. R. Glenn , S. J. DeVience , L. M. Pham , L. Rahn‐Lee , M. D. Lukin , A. Yacoby , A. Komeili , R. L. Walsworth , Nature 2013, 496, 486. 23619694 10.1038/nature12072 PMC3641584

[59] T. Rendler , J. Neburkova , O. Zemek , J. Kotek , A. Zappe , Z. Chu , P. Cigler , J. Wrachtrup , Nat. Commun. 2017, 8, 14701. 28317922 10.1038/ncomms14701 PMC5364376

[60] I. Fescenko , A. Jarmola , I. Savukov , P. Kehayias , J. Smits , J. Damron , N. Ristoff , N. Mosavian , V. M. Acosta , Phys. Rev. Res. 2020, 2, 023394. 33117992 10.1103/physrevresearch.2.023394 PMC7591154

[61] J. S. Sidhu , S. K. Joshi , M. Gündoğan , T. Brougham , D. Lowndes , L. Mazzarella , M. Krutzik , S. Mohapatra , D. Dequal , G. Vallone , P. Villoresi , A. Ling , T. Jennewein , M. Mohageg , J. Rarity , I. Fuentes , S. Pirandola , D. K. L. Oi , IET Quantum Commun. 2021, 2, 182.

[62] S. Frick , A. McMillan , J. Rarity , Opt. Express 2020, 28, 37118. 33379552 10.1364/OE.399902

[63] J. R. Johansson , P. D. Nation , F. Nori , Comput. Phys. Commun. 2012, 183, 1760.

