# Supplementary material for: Robotic Vectorial Field Alignment for Spin‐Based Quantum Sensors
Source: Adv Sci (Weinh). 2023 Nov 17;11(2):2304449. doi: 10.1002/advs.202304449 (PMC10787065; doi:10.1002/advs.202304449)
Supplement: Supplementary file 1 — Supporting Information [file ADVS-11-2304449-s001.pdf]

ADVANCED SCIENCE
Open Access

Supporting Information

for *Adv. Sci.*, DOI 10.1002/advs.202304449

Robotic Vectorial Field Alignment for Spin-Based Quantum Sensors

Joe A. Smith, Dandan Zhang and Krishna C. Balram**

Supporting Information

Robotic vectorial field alignment for spin-based quantum sensors.

Joe A. Smith,[1,*] Dandan Zhang,[2] and Krishna C. Balram[1,†]

[1]*Quantum Engineering Technology Labs and Department of Electrical and Electronic Engineering, University of Bristol, Bristol, BS8 1FD, UK*

[2]*Bristol Robotics Laboratory and Department of Engineering Mathematics, University of Bristol, Bristol, BS8 1TW, UK*

(Dated: October 24, 2023)

In this supplement we detail technical information to support the main text.

I. MAGNET FIELD STRENGTH

Neodymium permanent magnets, comprising of a neodymium-iron-boron alloy (NdFeB), are known for their high strength owing to exceptional uniaxial magnetocrystalline anisotropy and the high magnetic dipole moment of neodymium. These magnets are graded from N28-N55 based on their increasing strength. In the main text, we use N52 grade NdFeB owing to its high strength and availability, with a magnetic remanence of 1480 mT at 300 K [S1]. Using this value and its density of 7.5 g/cm^3, we calculate the field fall for different geometry magnets within the robot payload limit of 300 g in Maglibpy (described in the main text). In Fig. S1A, we observe that square prism magnets with a width of 2 cm have a surface magnetic field approaching 800 mT. We see a larger magnet face with 4 cm width has a lower surface field of 600 mT but exhibits a less steep roll off in field with distance from the magnet surface. In Fig. S1B, we see that cylindrical magnets, used in the experiments in the main text, have slightly lower but comparable magnetic fields for similar geometries. In general, the strength produced by these relatively small geometric footprint magnets make them highly attractive for application requiring field tunability in comparison to electromagnetic coils, which typically achieve a saturation field of 10-50 mT. In addition, the larger magnetic face chosen maintains a field uniformity of 99 % over millimetre regions, and would be therefore desirable for ensemble sensing applications where field uniformity over excitation volumes is desired.

II. ROBOT PARAMETERS

We use the Matlab Robotics Toolbox [S2] to analyze and visualize the robot's workspace and construct a model based on the Denavit-Hartenberg (DH) table of the robot, taking into account the specific joint limits (see Table S1). This model allows us to accurately represent the robot's structure and kinematics [S3]. By calculating the forward kinematics for sampled points between the joint limits, we determine the corresponding position and orientation of the robot's end effector in Cartesian space. In Fig S2B, we see this workspace

* j.smith@bristol.ac.uk

† krishna.coimbatorebalram@bristol.ac.uk

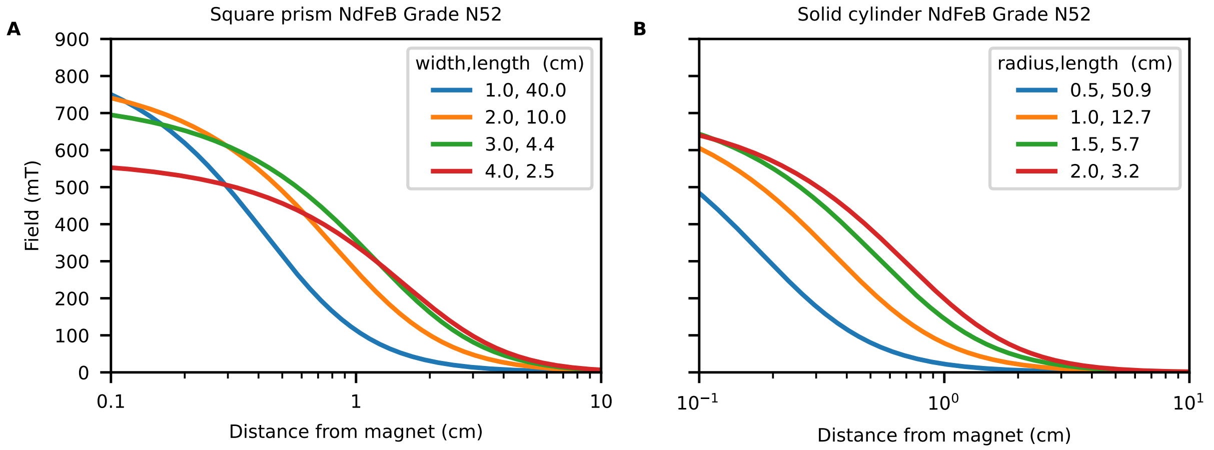

FIG. S1. **High field strength NeFeB magnets A.** Magnetic field strength for prism magnets with a square face set by the specified width, and magnetisation along the axial length. **B.** Magnetic field strength for cylindrical magnets with magnetisation along the axial length.

Link i	Twist angle $\alpha_{i-1}(^\circ)$	Length $a_{i-1}(m)$	Offset $d_i(m)$	Joint angle $\theta_i(^\circ)$	Joint limits $(^\circ)$
1	0	0.1663	0	θ_1	$[-169, 169]$
2	90	0	0	θ_2	$[-119, 34]$
3	0	0.221	0	θ_3	$[-78, 90]$
4	90	0.0325	0.235	θ_4	$[-119, 119]$
5	-90	0	0	θ_5	$[-105, 105]$
6	90	0	0.0285	θ_6	$[-150, 150]$

TABLE S1. Denavit-Hartenberg (DH) parameters of the robot used to define forward kinematics and the joint limits defining the maximum angular extent of the joints.

forms a complex 3D geometry approximately characterised by a 50 cm spherical volume about the world coordinate origin, with a unreachable 20 cm cylindrical centre. We deduce that the robot will produce axial magnetic fields, as described in Fig 1C of the main text, across trajectories with the end-effector contained between this inner and outer boundary.

For a full analysis, local performance indices can be calculated to quantify the dexterity of the robotic arm at points within the workspace. Here, we use the local manipulability

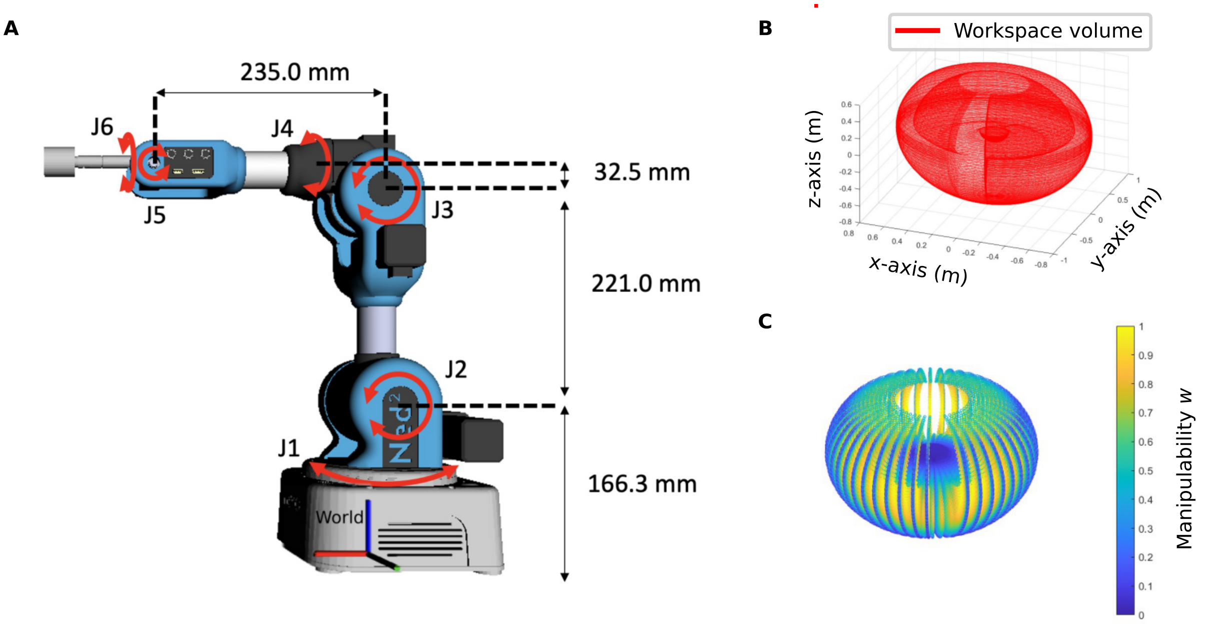

FIG. S2. **Robot parameters A.** Mechanical dimensions of the robot and joint rotational directions labelled J1-J6. World coordinate axis follows the main text (x-axis indicated in red, y-axis indicated in green, z-axis in blue). **B.** Reachable workspace volume of the robot, with the workspace boundary shown in red. **C.** Local manipulability, bounded by [0,1], as defined in the text with 1 indicating high manipulability.

measure w, defined as the scalar [S4]:

$$w = \sqrt{\det(\mathbf{J}\mathbf{J}^{\mathbf{T}})}, \tag{S1}$$

In Fig S2C, we evaluate w over the sampled points. This measure provides a quantitative assessment of the robot's ability to perform tasks within specific regions of its workspace, highlighting areas where the robot possesses higher or lower manipulability. We see that the robot maintains a high manipulability approaching unity within its reachable workspace.

III. CONFOCAL MICROSCOPE MEASUREMENTS

A schematic diagram of the scanning stage confocal setup described in the main text is given in Fig S3A. By synchronising the stage position with the detected photon counts, confocal scans are performed. The contrast in detected counts with applied MW frequency

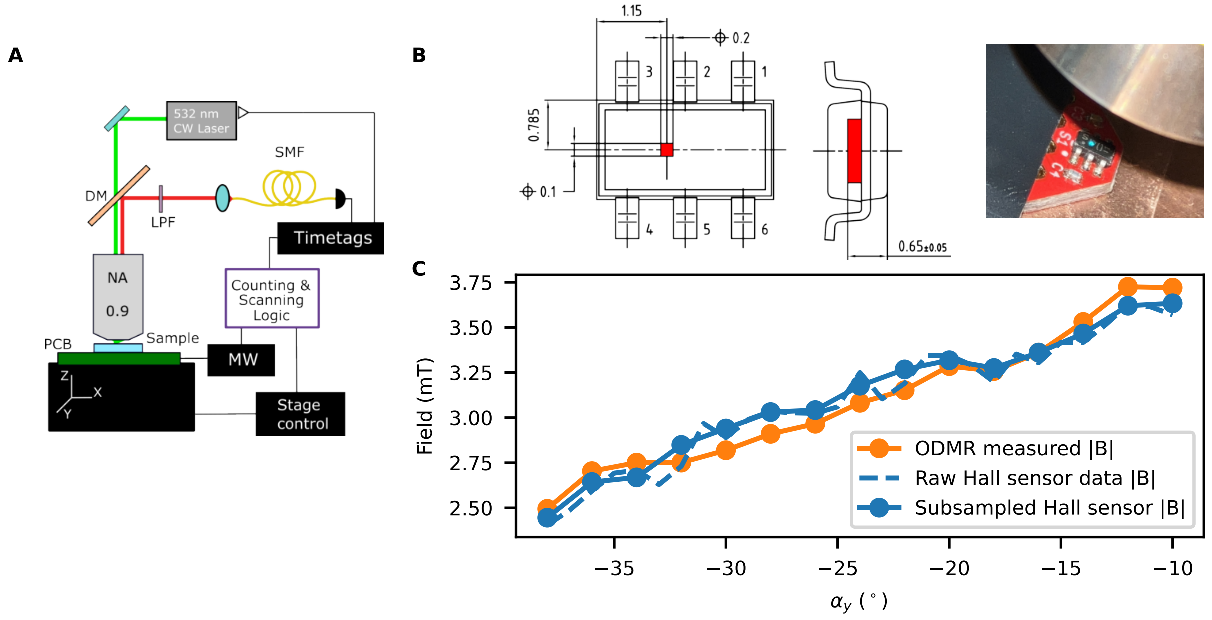

FIG. S3. **Confocal microscope measurements A.** Setup diagram. The NV centre is excited with a 532 nm Continuous Wave (CW) laser through a high Numerical Aperture (NA=0.9) objective. Emission is split from the pump using a Dichroic Mirror (DM) and Long Pass Filtered (LPF) before coupled into Single Moded Fibre (SMF) and detected. The microwave source (MW) radiates the sample through a PCB loop antenna. **B.** The Hall sensor is aligned with the NV centre by illuminating the sensitive region (in red) with the excitation beam (diagram units in mm). **C.** The ODMR data is well correlated with the Hall sensor measured field magnitude $|B|$.

forms the ODMR data in the main text. Experiment control and data collection is carried out using the QuDi framework [S5].

In order to co-align the position of the 3D Hall sensor and the NV centre, the stage is translated such that the sensitive region of the Hall sensor device (Fig S3B) is illuminated by the fixed objective lens. The z-position can be found by maximising the reflectivity of the excitation laser on the sample surface, and then translating the stage towards the objective by 500 µm such that the sensitive region is located at the same position as the aligned NV centre. We extract the field magnitude from the ODMR data presented in the main text using the formula presented in Balasubramanian *et al.* [S6]:

$$|B| = \frac{1}{\gamma}\sqrt{\frac{1}{3}\left(v_1^2 + v_2^2 - v_1 v_2 - D^2\right) - \Pi^2}, \tag{S2}$$

Technique	DOF	Estimated Volume (m)	Field	Further constraints	Ref.
Helmholtz coil	1-3	$0.5 \times 0.5 \times 0.5$	10 mT	Geometry	[S7]
Microcoil	1-3	$0.01 \times 0.01 \times 0.01$	10 mT	Heating	[S8]
Magnet on stage	1-3	$0.01 \times 0.1 \times 0.1$	1 T	Geometry, speed	[S9]
Electromagnet on stage	3	$0.01 \times 0.1 \times 0.1$	20 mT	Angular adjustment	[S10]
Hallbach array	1	$0.05 \times 0.1 \times 0.1$	1.5 T	Adjustability	[S11]
Superconducting coil	1-3	$1 \times 1 \times 1$	>5 T	Cost, cryogens	[S11]
Robotic system	5	$0.05 \times 0.05 \times 1$	1 T	Uniformity	(This work)

TABLE S2. A comparison of state-of-the-art techniques used to produce vector magnetic fields and align solid-state spins, including the technique described in this work. DOF here refers to the tunable Degrees of Freedom. Field refers to the reported magnetic field strength maximum.

with γ the gyromagnetic ratio, D and Π fitted from the zero-field splitting as before and v_1 and v_2 the two fitted resonant frequencies. In Fig. S3C, we find this extracted $|B|$ field in excellent agreement with the field amplitude directly measured by the Hall sensor. We subsample the Hall sensor field (as shown in S3C for Trajectory 2) for the common normalisation for Trajectory 1 and Trajectory 2 in Fig. 4G of the main text.

[S1] E Diez-Jimenez, JL Perez-Diaz, C Ferdeghini, F Canepa, C Bernini, C Cristache, J Sanchez-Garcia-Casarrubios, I Valiente-Blanco, Elisa María Ruiz-Navas, and JA Martínez-Rojas. Magnetic and morphological characterization of nd2fe14b magnets with different quality grades at low temperature 5–300 k. *Journal of Magnetism and Magnetic Materials*, 451:549–553, 2018.

[S2] Peter Corke. Robotics, vision and control: Fundamental algorithms in matlab. 2017.

[S3] Harvey Lipkin. A note on Denavit-Hartenberg notation in robotics. In *International Design Engineering Technical Conferences and Computers and Information in Engineering Conference*, volume 47446, pages 921–926, 2005.

[S4] Tsuneo Yoshikawa. Dynamic manipulability of robot manipulators. *Transactions of the*

Society of Instrument and Control Engineers, 21(9):970–975, 1985.

[S5] Jan M Binder, Alexander Stark, Nikolas Tomek, Jochen Scheuer, Florian Frank, Kay D Jahnke, Christoph Müller, Simon Schmitt, Mathias H Metsch, Thomas Unden, et al. Qudi: A modular python suite for experiment control and data processing. *SoftwareX*, 6:85–90, 2017.

[S6] Gopalakrishnan Balasubramanian, IY Chan, Roman Kolesov, Mohannad Al-Hmoud, Julia Tisler, Chang Shin, Changdong Kim, Aleksander Wojcik, Philip R Hemmer, Anke Krueger, et al. Nanoscale imaging magnetometry with diamond spins under ambient conditions. *Nature*, 455(7213):648–651, 2008.

[S7] Sebastian Knauer, John P Hadden, and John G Rarity. In-situ measurements of fabrication induced strain in diamond photonic-structures using intrinsic colour centres. *npj Quantum Information*, 6(1):50, 2020.

[S8] Huiliang Zhang, Keigo Arai, Chinmay Belthangady, J-C Jaskula, and Ronald L Walsworth. Selective addressing of solid-state spins at the nanoscale via magnetic resonance frequency encoding. *npj Quantum Information*, 3(1):31, 2017.

[S9] BD Wood, GA Stimpson, JE March, YND Lekhai, CJ Stephen, BL Green, AC Frangeskou, L Ginés, S Mandal, OA Williams, et al. Long spin coherence times of nitrogen vacancy centers in milled nanodiamonds. *Physical Review B*, 105(20):205401, 2022.

[S10] Matthias Widmann, Sang-Yun Lee, Torsten Rendler, Nguyen Tien Son, Helmut Fedder, Seoyoung Paik, Li-Ping Yang, Nan Zhao, Sen Yang, Ian Booker, et al. Coherent control of single spins in silicon carbide at room temperature. *Nature materials*, 14(2):164–168, 2015.

[S11] C Adambukulam, VK Sewani, HG Stemp, S Asaad, MT Madzik, A Morello, and A Laucht. An ultra-stable 1.5 t permanent magnet assembly for qubit experiments at cryogenic temperatures. *Review of Scientific Instruments*, 92(8), 2021.
